# Beyond *ℓ*_1_ sparse coding in V1

**DOI:** 10.1371/journal.pcbi.1011459

**Published:** 2023-09-12

**Authors:** Ilias Rentzeperis, Luca Calatroni, Laurent U. Perrinet, Dario Prandi

**Affiliations:** 1 Université Paris-Saclay, CNRS, CentraleSupélec, Laboratoire des Signaux et Systèmes, Paris, France; 2 CNRS, UCA, INRIA, Laboratoire d’Informatique, Signaux et Systèmes de Sophia Antipolis, Sophia Antipolis, France; 3 Aix Marseille Univ, CNRS, INT, Institut de Neurosciences de la Timone, Marseille, France; UT Austin: The University of Texas at Austin, UNITED STATES

## Abstract

Growing evidence indicates that only a sparse subset from a pool of sensory neurons is active for the encoding of visual stimuli at any instant in time. Traditionally, to replicate such biological sparsity, generative models have been using the *ℓ*_1_ norm as a penalty due to its convexity, which makes it amenable to fast and simple algorithmic solvers. In this work, we use biological vision as a test-bed and show that the soft thresholding operation associated to the use of the *ℓ*_1_ norm is highly suboptimal compared to other functions suited to approximating *ℓ*_*p*_ with 0 ≤ *p* < 1 (including recently proposed continuous exact relaxations), in terms of performance. We show that *ℓ*_1_ sparsity employs a pool with more neurons, i.e. has a higher degree of overcompleteness, in order to maintain the same reconstruction error as the other methods considered. More specifically, at the same sparsity level, the thresholding algorithm using the *ℓ*_1_ norm as a penalty requires a dictionary of ten times more units compared to the proposed approach, where a non-convex continuous relaxation of the *ℓ*_0_ pseudo-norm is used, to reconstruct the external stimulus equally well. At a fixed sparsity level, both *ℓ*_0_- and *ℓ*_1_-based regularization develop units with receptive field (RF) shapes similar to biological neurons in V1 (and a subset of neurons in V2), but *ℓ*_0_-based regularization shows approximately five times better reconstruction of the stimulus. Our results in conjunction with recent metabolic findings indicate that for V1 to operate efficiently it should follow a coding regime which uses a regularization that is closer to the *ℓ*_0_ pseudo-norm rather than the *ℓ*_1_ one, and suggests a similar mode of operation for the sensory cortex in general.

## Author summary

Recordings in the brain indicate that relatively few sensory neurons are active at any instant. This so called sparse coding is considered a hallmark of efficiency in the encoding of natural stimuli by sensory neurons. Computational works have shown that if we add sparse activity as an optimization term in a generative model encoding natural images then the model will learn units with receptive fields (RFs) similar to the neurons in the primary visual cortex (V1). Traditionally, computational models have used the *ℓ*_1_ norm as the sparsity term to be minimized, because of its convexity and claims of optimality. Here we show that by using sparsity inducing regularizers that approximate the *ℓ*_0_ pseudo-norm, we get sparser activations for the same quality of encoding. Moreover, for a certain level of sparsity, both *ℓ*_0_ and *ℓ*_1_ based generative models produce RFs similar to V1 biological neurons, but the *ℓ*_1_ model has five times worse encoding performance. Our study thus shows that sparsity-inducing regularizers approaching the *ℓ*_0_ pseudo-norm are more appropriate for modelling biological vision from an efficiency point of view.

## Introduction

Sensory neurons produce a variable range of responses to stimuli, the most frequent one being inactivity [1, 2]. To explain this, Horace B. Barlow hypothesized that the task of sensory neurons is not only to encode in their activity an accurate representation of the outside world, but to do so with the least possible number of active neurons at any time [3]. Since then, growing experimental evidence across species and sensory areas has confirmed these claims of sparse activity [4–8].

Using Barlow’s hypothesis as an optimization principle, Olshausen and Field showed that a neural network equipped with a learning algorithm that is set to reconstruct natural images with sparse activity constraints develops units with properties similar to the ones of receptive fields (RFs) of simple cells in the primary visual cortex (V1), i.e. they are bandpass, oriented and spatially localized [9]. The model proposed by the authors belongs to the family of generative algorithms which represent a stimulus as a linear combination of units taken from an overcomplete dictionary, i.e. a set of vectors with more basis vectors than the dimension of the stimuli. In the context of V1, the vectors from the dictionary and their accompanying coefficients correspond to the neurons’ RFs and activities, respectively.

Computationally, overcompleteness comes with a number of advantages: the input can be in a compressed form [10], the emerging vectors in the overcomplete dictionary are shiftable, and transformations of the input image such as rotations or translations can be represented by smooth changes in the coefficients [11]. Experimental findings in the macaque show that overcomplete dictionaries reflect the expansion of neurons in layer 4C*α* of V1 compared to the lateral geniculate nucleus (LGN) input to that area; approximately, 30 LGN neurons send their axons to a V1 hypercolumn containing about 3000 excitatory and 1000 inhibitory neurons [12–14].

A sparse approximation aims to find a linear combination of the dictionary vectors that has few nonzero coefficients but also adequately represents the input signal. Ideal sparse approximation requires the minimization of the noncontinuous and nonconvex *ℓ*_0_ pseudo-norm, which counts the number of nonzero coefficients, combined with some data fitting term. However, this problem is NP-hard, as its solution requires an intractable combinatorial search [15, p.418]. Greedy pursuit methods are practical solutions which bear resemblance to neural spiking processes [16], yet their efficiency can be improved. In many applications, the *ℓ*_0_ pseudo-norm has been replaced with its convex relaxation, the *ℓ*_1_ norm, defined as the sum of the absolute values of the coefficients. The use of the *ℓ*_1_ relaxation has become widespread in sparse coding, due to its convexity, and since under certain conditions [17, 18] solutions of the *ℓ*_1_-penalized sparse coding problem coincide with the ones making use of *ℓ*_0_ regularization. In general, however, *ℓ*_1_-based models show inferior results in terms of sparsity [19, 20].

Over the last decade, advances in optimization theory and in the field of compressed sensing [21] have provided several tools allowing for the replacement of the *ℓ*_0_ pseudo-norm with tractable functions approximating it. The use of such approaches provides solutions for many perceptual and behavioral tasks that are in line with the energy constraints in the brain, unlike exact solutions that need perfect prior knowledge and costly computations [22].

In this work, we examined different sparse coding algorithms relying on the use of tighter thresholding functions associated to the use of *ℓ*_*p*_ penalties, with 0 ≤ *p* < 1. We found that their solutions induce sparsity to a greater extent compared to the *ℓ*_1_ method (soft thresholding, also called ISTA) while they maintain the same reconstruction of the signal. As a further penalty we used the Continuous Exact *ℓ*_0_ relaxation (CEL0) [23] which produced the sparsest codes.

We then analyzed the RFs learned by the resulting sparse coding algorithms and compared them with each other and with the RFs found in the visual cortex of non-human primates. We found that all algorithms yield localized oriented RFs. As we increased the degree of overcompleteness, we found that most units shifted from sharp orientation selectivity to a broader one. In a setting where different sets of neurons with variable numbers act as separate modules (with different degrees of overcompleteness each) reconstructing in their totality many times over the external world, the generative model could explain the broad orientation selectivity of neurons in V1 [24, 25]. In accordance with the oblique effect and its representation in the visual cortex [26, 27], we found a preference towards the vertical orientation both in terms of overrepresentation and increased sensitivity of RFs tuned to it.

In terms of performance, when keeping sparsity constant for all methods, we found that soft thresholding requires a dictionary of 10 times more units to reconstruct the input image patch as well as CEL0. The other methods considered, relying, e.g., on *ℓ*_1/2_ minimization [28] and on hard thresholding [29] are inferior to CEL0 in terms of reconstruction performance but still superior to soft thresholding.

By definition, *ℓ*_1_ regularization employed by soft thresholding limits the absolute sum of activations rather than the number of active neurons [9, 30]. Recent results on the metabolic expenditure of neurons have indicated that a regime with few neurons firing vigorously (akin to *ℓ*_0_ regularization) is far more energy efficient than one with more neurons firing at a lower rate (*ℓ*_1_ regularization) [31]. This is corroborated by a recent study on mice showing that natural images could be decoded from a very small number of highly active V1 neurons, and that diverse RFs ensure both reliable and sparse representations [32].

In our work we show that at a specific sparsity level both *ℓ*_0_-type and *ℓ*_1_ regularizers can learn RF shapes similar to V1 biological cells [33], mostly round or slightly elongated. But, for this sparsity level, CEL0 has approximately five times better reconstruction performance of the external stimulus compared to the *ℓ*_1_ regularizer (ISTA). Our results indicate that *ℓ*_0_ based regularization is more appropriate for the visual cortex to operate efficiently.

## Materials and methods

### Image dataset and preprocessing

From the van Hateren’s database [34], we used for our tests a selection of 137 natural images that did not contain artificially created structures neither significant blur [35]. We performed the same preprocessing stage described in [36]: first, we rescaled all images separately between zero and one, then we normalized them by subtracting and dividing each pixel value by the image mean and standard deviation respectively. The resulting zero mean, unit variance images were then passed through a whitening filter in order to emulate the response of retinal ganglion cells. The images were finally rescaled such that they have a variance of 0.1. This value serves as a baseline error, i.e. the mean square error (MSE) of a preprocessed image with an image with only zero pixel values (produced when all the coefficients of a neural code are zero). S1 Fig shows examples of raw and preprocessed images.

### Sparse coding generative models

#### Model setup and cost function

According to the linear generative model of Olshausen and Field [9], an image $I\in R^{M}$ is described as a linear combination of vectors ${\left(\phi_{i}\right)}_{i=1}^{N}$ with $\phi_{i}\in R^{M}$ for all *i*. The vectors ${\left(\phi_{i}\right)}_{i=1}^{N}$ are stored column-wise in a matrix $\Phi \in R^{M\times N}$. The scalar coefficients of such linear combination are collected in a vector $r\in R^{N}$ and an additive white Gaussian noise component $\nu \in R^{M}$ with $\nu ∼N(0,\sigma^{2}\text{Id})$ is added to model perturbations and uncertainty:

$$\begin{array}{c} I=\Phi r+\nu =\sum_{i=1}^{N}r_{i}\phi_{i}+\nu . \end{array}$$ (1)

We consider features (columns) of Φ to form an overcomplete dictionary, i.e. *N* ≫ *M*. Consequently, the inverse problem of finding *r* given *I* in (1) becomes ill-posed since *r* may have an infinite number of possible solutions. To impose well-posedness, Olshausen and Field [9] considered a sparse regularisation approach, defined in terms of the energy function:

$$\begin{array}{c} E\left(r,\Phi \right)≔\frac{1}{2}{\left\|I-\Phi r\right\|}_{2}^{2}+\lambda \cdot \sum_{i=1}^{N}c\left(r_{i}\right). \end{array}$$ (2)

While the first term in (2) pushes towards the preservation of stimulus information, the second term acts as regularization imposing a penalty on activity with the relative weight of the two competing tasks being controlled by a parameter λ > 0. The regularization term $C\left(r\right)≔\sum_{i=1}^{N}c\left(r_{i}\right)$ is a sparsity-promoting penalty that ideally encourages the number of active units to be as few as possible. For that, one would like to choose as *c*(⋅) the so-called *ℓ*_0_ pseudo-norm of *z* which costs 1 whenever *z* ≠ 0 and 0 otherwise: *c*(*z*) = ‖*z*‖_0_, with $z\in R^{N}$. However, as shown rigorously in several mathematical works (e.g., [37]) such choice makes the problem of minimising *E* in (2) NP-hard. A standard strategy, used in several sparse coding approaches, relies on the use of the convex and continuous *ℓ*_1_ norm as a relaxation, i.e., *c*(*z*) = |*z*| for z∈R. Under suitable conditions on the matrix Φ, such choice guarantees indeed that the solution computed is equivalent to the one corresponding to the *ℓ*_0_ pseudo-norm. The use of the *ℓ*_1_ norm is in fact established in the field of compressed sensing and sparse signal/image processing [38].

For general choices of *c*(⋅), the problem of finding both optimal sparse codes r* (coding step) and feature vectors Φ* for the given input stimulus *I* (learning step) can be formulated as the problem of minimizing the energy function *E* with respect to both *r* and Φ, i.e:

$$\begin{array}{c} \left(r^{*},\Phi^{*}\right)\in \underset{r\in R^{N},\Phi \in R^{M\times N}}{\text{arg}\;\text{min}}\;E\left(r,\Phi \right). \end{array}$$ (3)

In the following, we use an alternating minimization (see, e.g., [39]) to solve the problem above. Below, we thus make precise the general approach for solving the coding and learning steps. Subsequently, we consider few cost functions promoting sparsity in different ways.

#### Coding step

Our objective is to minimize the composite function *E*(*r*, Φ) in (2) which is defined as the sum

$$\begin{array}{c} E(r,\Phi )=f(r,\Phi )+\lambda C(r), \end{array}$$ (4)

with $f:R^{N}\times R^{M\times N}\to R_{+}$ being convex and differentiable with *L*-Lipschitz gradient w.r.t. both variables and $C:R^{N}\to R_{+}$ being convex, proper and lower semi-continuous, but, generally, non-smooth. As far as the coding step is concerned, we then need an algorithm solving the structured nonsmooth optimisation problem (3) w.r.t. *r*. A standard strategy for this is to use the proximal gradient algorithm (see [40] for a review). For a given step-size *μ* ∈ (0, 1/*L*], $x_{0}\in R^{N}$, $\bar{\Phi }\in R^{M\times N}$ and *t* ≥ 0, such algorithm consists in the alternative application of the two steps:

- Gradient step: $r_{t+1}=r_{t}-\mu \nabla f\left(r_{t},\bar{\Phi }\right)$;
- Proximal step: *r*_*t*+1_ = prox_*μ*,λ*C*_(*r*_*t*+1_),

where the proximal operator associated to the function λ*C*(⋅) and depending on the step-size parameter *μ* is defined by:

$$\begin{array}{cc} {\text{prox}}_{\mu ,\lambda C}\left(z\right) & =\underset{y\in R^{N}}{\text{arg}\;\text{min}}\;\lambda C\left(y\right)+\frac{1}{2\mu }{\left\|y-z\right\|}^{2}\\ & =\underset{y\in R^{N}}{\text{arg}\;\text{min}}\;C\left(y\right)+\frac{1}{2\lambda \mu }{\left\|y-z\right\|}^{2}\\ & ={\text{prox}}_{1,\mu \lambda C}\left(z\right),\;z\in R^{N}. \end{array}$$ (5)

It is common to denote by *T*_*μ*λ_(*z*) the thresholding operation corresponding to prox_*μ*,λ*C*_(*z*), which sets to zero the components of *z* which are too large depending on a certain thresholding rule defined in terms of the choice of *C* and the thresholding parameter *μ*.

Note that during coding, the vectors in $\bar{\Phi }$ are kept fixed, so that the algorithm seeks the optimal activations for the given input image patches.

#### Learning step

During learning, the matrix $\Phi \in R^{M\times N}$ is updated so that it is optimal in reconstructing the input *I* as accurately as possible. The learning step is thus obtained by minimizing (2) w.r.t. Φ, by considering gradient-type iterations. This step is easier since Φ appears only in the smooth data fit term and not in the cost term.

Learning is then obtained for all *t* ≥ 0 via the iterative procedure:

$$\begin{array}{c} \Phi_{t+1}=\Phi_{t}+\eta r\left(I-\Phi_{t}r\right), \end{array}$$ (6)

where *η* > 0 is the learning rate whose size has to be small enough to guarantee convergence. Note that, although such update of Φ does not depend explicitly on the particular choice of the cost function considered, it depends nevertheless on the current estimate of the coefficients *r* which, in turn, depend on the particular choice of *C* and, consequently, *T*_*μ*λ_. During each learning step, we impose the norms of the current iterate Φ_*k*_ to be equal to 1, though other normalization mechanisms could be explored [41].

### Thresholding operators

**Fig 1:**
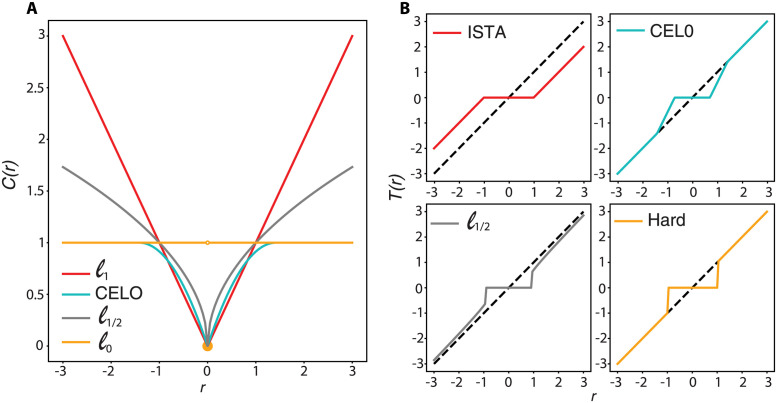
Sparsity-promoting cost functions *c* considered and their corresponding thresholding operators. A: Plot of 1D cost functions $c:R\to R_{+}$ considered. B: Corresponding thresholding operators.

For different choices of the component-wise cost functions $c:R\to R_{+}$, different thresholding rules are derived. We consider below some particular choices of *c*, plotting them in Fig 1A for comparison. For each choice, we then report the corresponding explicit thresholding operator which sets to zero coefficients with small magnitudes, see Fig 1B for an illustration. As a technical note, we remark that in definition (5) we assumed the function *C* to be convex, so that the minimizer of the functional is uniquely defined due to the strong convexity of the composite function. A large class of the cost functions considered below, however, are not convex, hence definition (5) still holds, but with a ∈ sign in place of the equality one, since the set of minimizers may not be a singleton.

#### The iterative soft thresholding algorithm (ISTA)

For *c*(*r*_*i*_) = |*r*_*i*_| for all *i* = 1…, *N* sparsity in (2) is obtained by considering as regulariser the function *C*(*r*) = λ‖*r*‖_1_ which, from an algorithmic viewpoint, corresponds to the iterative soft thresholding algorithm (ISTA) as an algorithmic solver [29, 42]. Thanks to the separability of the *ℓ*_1_ norm, the proximal operator can be computed component-wise [43]. Setting *θ* ≔ *μ*λ > 0 and *S*_*θ*_(⋅) ≔ *T*_*θ*_(⋅), it holds that for all *i* = 1, …, *N*:

$$\begin{array}{c} S_{\theta }\left(r_{i}^{*}\right)=\{\begin{array}{cc} r_{i}^{*}-\theta & \left(r_{i}^{*}>\theta \right)\\ 0 & \left(-\theta \leq y\leq \theta \right)\\ r_{i}^{*}+\theta & (r_{i}^{*}<-\theta ). \end{array} \end{array}$$ (7)

Such operation is typically known in literature under the name of soft thresholding operator due to its continuity outside the vanishing thresholding region.

#### The iterative half thresholding algorithm

To favour more sparsity than the *ℓ*_1_ norm, a natural improvement consists in using the *ℓ*_*p*_ (0 < *p* < 1) pseudo-norm, i.e. setting *c*(*r*_*i*_) = |*r*_*i*_|^*q*^. However, such choice makes the optimization problem (2) nonsmooth and nonconvex and, in general, prevents from using fast optimisation solvers. One exception to this is the case when *ℓ*_1/2_ regularization is used. Xu and colleagues showed in [28] that an iterative half thresholding algorithm can solve the problem of minimising the *ℓ*_1/2_ pseudo-norm with an *ℓ*_2_ data fit term with the algorithm converging to a local minimizer in linear time [44]. Using analogous notation *θ* = λ*μ* as before and denoting by Ξ_*θ*,1/2_(⋅) the thresholding operator *T*_*θ*_, thresholding can still be performed component-wise as follows:

$$\begin{array}{c} \Xi_{\theta ,1/2}\left(r_{i}^{*}\right)=\{\begin{array}{cc} f_{\theta ,1/2}\left(r_{i}^{*}\right), & |r_{i}^{*}|>\sqrt[{3}]{54}\;\theta^{2/3}\\ 0, & \text{otherwise}, \end{array} \end{array}$$ (8)

where:

$$\begin{array}{c} f_{\theta ,1/2}\left(r_{i}^{*}\right)=\frac{2}{3}r_{i}^{*}(1+\text{cos}\;(\frac{2\pi }{3}-\frac{2}{3}\psi_{\theta }\left(r_{i}^{*}\right))) \end{array}$$ (9)

and

$$\begin{array}{c} \psi_{\theta }\left(r_{i}^{*}\right)=\text{arccos}(\frac{\theta }{8}{\left(\frac{|r_{i}^{*}|}{3}\right)}^{-3/2}). \end{array}$$ (10)

Despite its complex form, the thresholding function (8) is still explicit, hence its computation is very efficient.

#### The iterative hard thresholding algorithm

The iterative hard thresholding algorithm has been introduced firstly in [29] to overcome the NP-hardness associated to the minimization of the ideal problem:

$$\begin{array}{c} \underset{r\in R^{N}}{\text{arg}\;\text{min}}\;E_{\ell_{0}}\left(r\right)=\|I-\bar{\Phi }{r\|}_{2}^{2}+\lambda {\left\|r\right\|}_{0}, \end{array}$$ (11)

for $\bar{\Phi }\in R^{M\times N}$. The idea consists in considering the following surrogate function defined for $r,z\in R^{N}$ as:

$$\begin{array}{c} E_{\ell_{0}}^{S}\left(r,z\right)≔\|I-\bar{\Phi }{r\|}_{2}^{2}+{\lambda \|r\|}_{0}-\|\bar{\Phi }r-\bar{\Phi }{z\|}_{2}^{2}+{\left\|r-z\right\|}_{2}^{2}, \end{array}$$ (12)

for which there trivially holds $E_{\ell_{0}}\left(r\right)=E_{\ell_{0}}^{S}\left(r,r\right)$ for all $r\in R^{N}$. One can show that (12) is a majorizing functional for (11), that is, for a given $\bar{z}\in R^{N}$, $E_{\ell_{0}}\left(r\right)\leq E_{\ell_{0}}^{S}\left(r,\bar{z}\right)$ for all $r\in R^{N}$ and there holds $E_{\ell_{0}}\left(\bar{z}\right)=E_{\ell_{0}}^{S}\left(\bar{z},\bar{z}\right)$. In other words, minimizing (12) with respect to *r* can thus be seen as a strategy to minimize (11) by choosing at each step *t* of the iterations $\bar{z}=r^{t}$, that is the estimate of the desired solution at the previous step. The derived thresholding operator is here denoted by *H*_*θ*_(⋅) and performs element-wise hard thresholding following the rule:

$$\begin{array}{c} H_{\theta }\left(r_{i}^{*}\right)=\{\begin{array}{cc} 0, & |r_{i}^{*}|\leq \sqrt{\theta }\\ r_{i}^{*}, & \text{otherwise}. \end{array} \end{array}$$ (13)

#### A continuous exact *ℓ*_0_ penalty (CEL0)

As a further sparsity-promoting regularization, we consider the non-convex Continuous Exact relaxation of the *ℓ*_0_ pseudo-norm (CEL0), thoroughly studied, e.g., in [23]. Such choice can be thought of (as it is rigorously proved in [45]) as the inferior limit of the class of all continuous and non-convex regularizations of the *ℓ*_0_ pseudo-norm with the interesting additional properties of preserving the global minimizers of the ideal *ℓ*_2_-*ℓ*_0_ minimization problem one would need to solve, while reducing the number of the local ones. For all *i* = 1, …, *N* and parameter λ > 0 such choice corresponds to considering as cost functional:

$$\begin{array}{c} C_{\text{CEL0}}\left(r\right)≔\sum_{i=1}^{N}c(\|\phi_{i}\left\|,\lambda ,r_{i}\right)=\sum_{i=1}^{N}(\lambda -\frac{\|\phi_{i}{\|}^{2}}{2}{\left(|r_{i}|-\frac{\sqrt{2\lambda }}{\|\phi_{i}\|}\right)}_{+}^{2}), \end{array}$$ (14)

where *ϕ*_*i*_ is the *i*-th column extracted from the matrix Φ and, for all z∈R, the notation (*z*)_+_ denotes the positive part of *z*, i.e. (*z*)_+_ = max(0, *z*). The corresponding CEL0 thesholding operator is defined by:

$$\begin{array}{c} \Theta_{\mu ,\lambda }^{\text{CEL0}}\left(r_{i}^{*}\right)=\{\begin{array}{cc} \text{sign}\left(r_{i}^{*}\right)\;\text{min}\;\{|r_{i}^{*}|,\frac{\left(\right|r_{i}^{*}|-\sqrt{2\lambda }\mu \|\phi_{i}{\left\|\right)}_{+}}{1-\|\phi_{i}{\|}^{2}\mu }\}, & \|\phi_{i}{\|}^{2}\mu <1\\ r_{i}^{*}1_{|r_{i}^{*}|>\sqrt{2\mu \lambda }}\left(r_{i}^{*}\right)+\left\{0,r_{i}^{*}\right\}1_{|r_{i}^{*}|=\sqrt{2\mu \lambda }}\left(r_{i}^{*}\right), & \|\phi_{i}{\|}^{2}\mu \geq 1, \end{array} \end{array}$$ (15)

where, note, *μ* and λ are here decoupled as the thresholding parameter is not their parameter anymore but depends on *μ* only and, component-wise, by the norm of the column *ϕ*_*i*_, *i* = 1, …, *N* of Φ. While the operation of computing the quantities ‖*ϕ*_*i*_‖ can be in principle costly from a computational viewpoint, we remark that by construction, in our application Φ has unit-norm columns, hence such computation is in fact not required.

### A measure for orientation selectivity: Circular variance

To probe the orientation selectivity of each ${\left(\phi_{i}\right)}_{i=1}^{N}$ vector (unit) estimated by the different models above as well as to compare them with each other and with experimental data in V1, we used the circular variance measure (*V* ∈ [0, 1]) [24, 46, 47]. A unit with a zero circular variance responds only to a particular orientation; a unit with a circular variance of one responds to all orientations equally. Values in between show some selectivity, with the ones closer to one showing a broader orientation selectivity compared to the ones closer to zero.

The circular variance of a vector *ϕ* is defined as *V* ≔ 1 − |*R*| where *R* is:

$$\begin{array}{c} R=\frac{\sum_{k}\alpha_{k}e^{i2\theta_{k}}}{\sum_{k}\alpha_{k}}, \end{array}$$ (16)

with *α*_*k*_ being the response of the unit at the orientation *θ*_*k*_ (*θ*_*k*_ goes from 0 to *π* in *k* = 36 equidistant steps). A plot of *α*_*k*_ as a function of *θ*_*k*_ for a unit corresponds to its orientation tuning curve.

We get the *α*_*k*_ values for all *θ*_*k*_ orientations for a unit *ϕ*_*i*_ by first finding the spatial frequency for which the unit responds the most. We do that by taking the inner product of *ϕ*_*i*_ with a bank of sinusoidal gratings of different spatial frequencies, orientations, and phases. The grating giving the highest inner product value yields the optimal spatial frequency. We subsequently narrow down the gratings that we test, to the ones with the optimal spatial frequency. To then get the unit’s orientation tuning curve (*α*_*k*_ as a function of *θ*_*k*_), we use for each orientation the highest value from the inner products of *ϕ*_*i*_ with the subset of gratings of the same orientation but different phase. We then proceed in estimating the circular variance for unit *ϕ*_*i*_ from Eq (16). Examples of RFs generated by the thresholding algorithms and their corresponding orientation tuning curves produced as outlined here are shown in S2 Fig.

We aim in the Results section to compare the sparse coding obtained by the different choices of the thresholding functions. More specifically, we probe the relationship between sparsity level and reconstruction performance at different dictionary sizes for each algorithm. Moreover, as the coding step affects the learning step, we examine how the choice of the sparsity-promoting penalty affects the estimation of the RFs *ϕ* at convergence. Previously, it has been shown that a highly overcomplete dictionary, or a very sparse code —for a dictionary with fixed units— produces RFs with different functionalities [48, 49]. In the following tests, we probe whether the different algorithms considered generate RFs that are close to biological ones.

## Results

### Sparsity of codes

**Fig 2:**
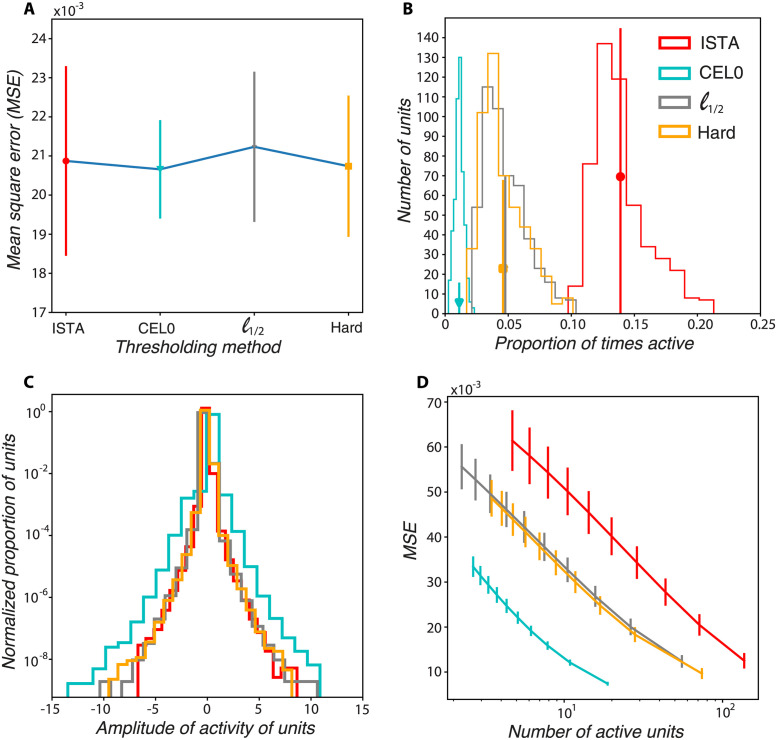
CEL0, and to a lesser extent, *ℓ*_1/2_, and hard thresholding produce sparser codes than ISTA. A: MSE between the reconstructed and the actual image for the last 500 batches as a function of the thresholding method. B: Distribution of activity of the units for the image stimuli presented. The middle of the vertical lines represent the mean number of active units per image patch, their length the standard deviation. C: Distribution of the amplitudes of the active units D:MSE as a function of the number of active units. To get these data points we varied the λ parameter for each algorithm.

We first compared the sparsity of the codes produced by the different thresholding algorithms, each containing 500 units (∼ 2× degrees of overcompleteness). To make a fair comparison, we adjusted the methods’ parameters λ and *μ* so that they all produce reconstructed images with the same MSE (the values of the parameters are shown in S1 Table). We run the algorithms for 4000 batches, with each batch containing 250 image patches. In all cases, the MSE for the last 500 batches is about 0.021 (see Fig 2A for the mean values, and S3 Fig for the evolution of the MSE as a function of iterations, i.e. as we learn the *ϕ* vectors). As expected from preprocessing, the baseline error, i.e. the MSE corresponding to the zero image when all units’ activations are zero, is 0.1. We found that for the same MSE, CEL0, *ℓ*_1/2_, and hard thresholding algorithms produce sparser codes compared to ISTA, with *ℓ*_1/2_, and hard thresholding having very similar activity distributions and CEL0 being the approach corresponding to the sparsest solutions (Fig 2B). As expected, since ISTA aims to minimize the sum of the activations, its units’ amplitude distribution has a smaller variance (spread from zero) compared to the other methods (Fig 2C). We varied the λ parameter for all four thresholding algorithms so that we test whether the sparsity difference between them holds in general. We found that consistently, ISTA needs more active units to achieve the same reconstruction performance as the other methods, with CEL0 providing the sparsest solutions (Fig 2D).

The vectors ${\left(\phi_{i}\right)}_{i=1}^{N}$ are updated (learning step) at each iteration on a batch of image patches. The different thresholding algorithms produce vectors *ϕ* that are not alike. We thus asked whether the different thresholding algorithms considered have similar coding performances—in terms of sparsity and reconstruction error—when they used a set of vectors *ϕ* that is different from the one they would normally learn. We probed this question by using throughout the coding steps a fixed dictionary Φ (we considered, in particular all dictionaries at convergence of all four algorithms). We observed a sort of invariance property with respect to the dictionary used: all thresholding algorithms show similar reconstruction error and distributions of activity independently of the dictionary used in coding (S4 Fig). This suggests that the cost function landscape for learning the dictionaries has different local minima that are equally optimal.

**Fig 3:**
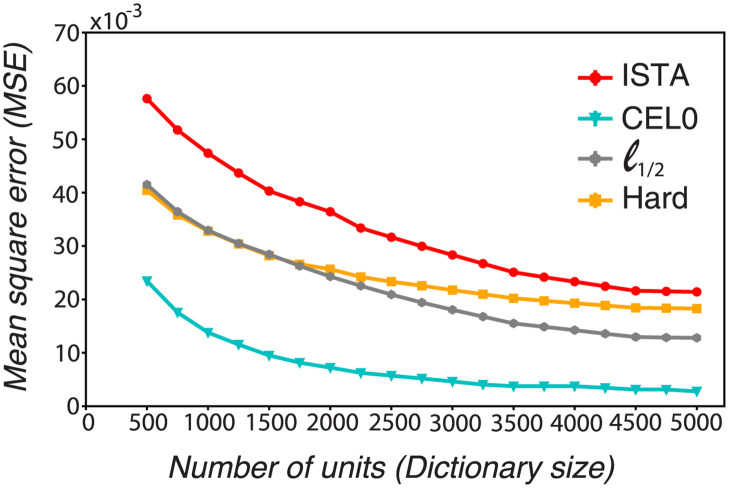
CEL0 and, to a lesser extent, *ℓ*_1/2_, and hard thresholding have better reconstruction error than ISTA for all dictionary sizes tested (from approximately 2 to 20 degrees of overcompleteness). MSE of the different thresholding methods for several dictionary sizes. The parameter λ has been adjusted for each dictionary size and algorithm so that the sparsity level is approximately stable (see S5 Fig). Each time we run 1600 batches of 250 image patches (in total 400000 patches), and took the mean and standard deviation of the reconstruction error of the last 100 batches.

We probed the reconstruction performance of the thresholding algorithms as we increased their degree of overcompleteness, i.e. the number of units, while keeping the level of sparsity relatively constant (see S5 Fig for the sparsity levels of the algorithms for different dictionary sizes, and S2 Table for the parameters values for the different methods and number of units). We found that the *ℓ*_1_ model needs approximately 5000 units to reach the reconstruction performance of CEL0 for 500 units (Fig 3). We also observed that for dictionary sizes greater than 2000 units (i.e., greater than 8× degrees of overcompleteness) *ℓ*_1/2_ thresholding has a smaller reconstruction error than hard thresholding, with both performing better than ISTA for any dictionary size. CEL0 provides consistently the best reconstruction performance.

### Learned dictionaries and their similarity with V1

#### Orientation selectivity of RFs

**Fig 4:**
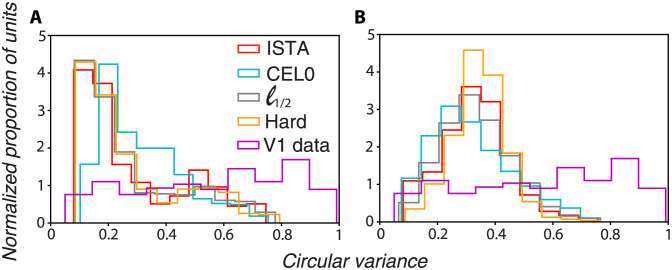
In contrast to macaque V1 neurons that have a uniform circular variance distributions, the units of all thresholding algorithms show a distinct peak in their circular variance distribution that shifts to the right (more broadly tuned neurons) as the units in the dictionary increase. Distribution of circular variance values for the *ϕ* learned by the different thresholding algorithms (the area in all cases was normalized to sum to 1) with V1 experimental data from [24] included for A: 500 units and B: 2000 units.

Experimental evidence indicates that neurons in V1 show great variability in their orientation selectivity: some neurons respond to a narrow band around a particular orientation, but most of them are responsive to a broader spectrum of orientations [24, 25]. To examine the orientation selectivity of the vectors *ϕ* produced by each thresholding algorithm and draw comparisons with experimental data, we used the circular variance measure ([24, 46, 47]). For this and subsequent analyses, unless otherwise stated, we use 500 units with the parameters shown in S1 Table). We found that all thresholding algorithms show a similar distribution with a peak at low circular variance values (sharp orientation selectivity), with the only minor difference being that the peak of the CEL0 distribution is slightly shifted to higher circular variance values (Fig 4A). Populations of V1 neurons in the macaques do not show a sharp peak for low circular variance values but rather a more uniform distribution across small and large values (Fig 4A; data taken from [24]). We subsequently asked whether the orientation tuning distribution generated by the models is dependent on their degree of overcompleteness. To answer that, we performed the same analysis for 2000 units (∼ 8× degrees of overcompleteness). We adjusted the λ parameter for CEL0 so that it will have the same sparsity as in the 500 units case; we tuned λ for the rest of the methods so that they have the same MSE as CEL0. We found that the orientation tuning curves become more broad as indicated by a shift in the peaks of the circular variance histograms to the right with CEL0’s histogram being more flattened (Fig 4B). Our results indicate that as the degree of overcompleteness of the units increase, their RFs become on average more broad. For both degrees of overcompleteness, however, the distributions show a distinct peak, in contrast to the more homogeneous distribution shape of the experimental data. Assuming that the experimental data is an unbiased sample of V1 simple neurons, our results show that the generative model, irrespective of the regularization used, produces units that correspond to a subset of the actual V1 neurons.

**Fig 5:**
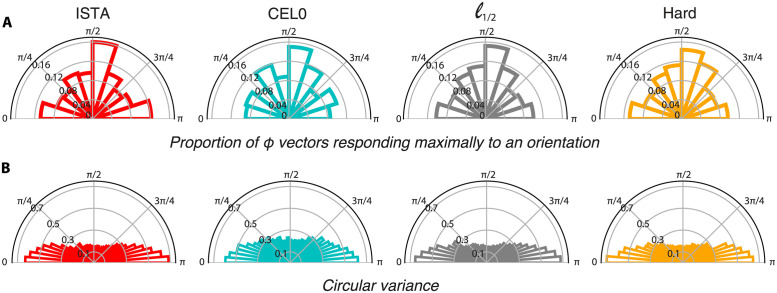
The largest number of *ϕ* vectors responding maximally to a particular orientation are the ones with a preference towards the vertical orientation, with this subset also showing the sharpest orientation tuning (as indicated by their circular variance). A: Polar plots of the proportion of *ϕ* vectors responding maximally to an orientation for ISTA, CEL0, *ℓ*_1/2_, and hard thresholding. B: Polar plots of the mean circular variance of *ϕ* vectors binned according to their preferred orientation.

Perceptually, visual stimuli are better resolved when they are presented in the cardinal orientations —either horizontal or vertical— as opposed to oblique ones [50]. This behavioural oblique effect has been suggested to emerge in part due to the over-representation of simple cells in V1 that respond to cardinal orientations as shown by single unit recordings [26, 27], optical imaging [51] and fMRI [52]. Moreover, single unit recordings indicate that cardinal orientations have narrower orientation tuning curves [27]. Our results for 500 units agree with the experimental findings most prominently for the vertical orientation (*π*/2). We find that the proportion of vectors *ϕ* responding maximally to the vertical orientation is the highest compared to all the other orientations (Fig 5A), and that this subset has vectors with the narrowest orientation tuning curves as indicated by their circular variance values (Fig 5B).

#### Sparsity-induced variability of RFs

**Fig 6:**
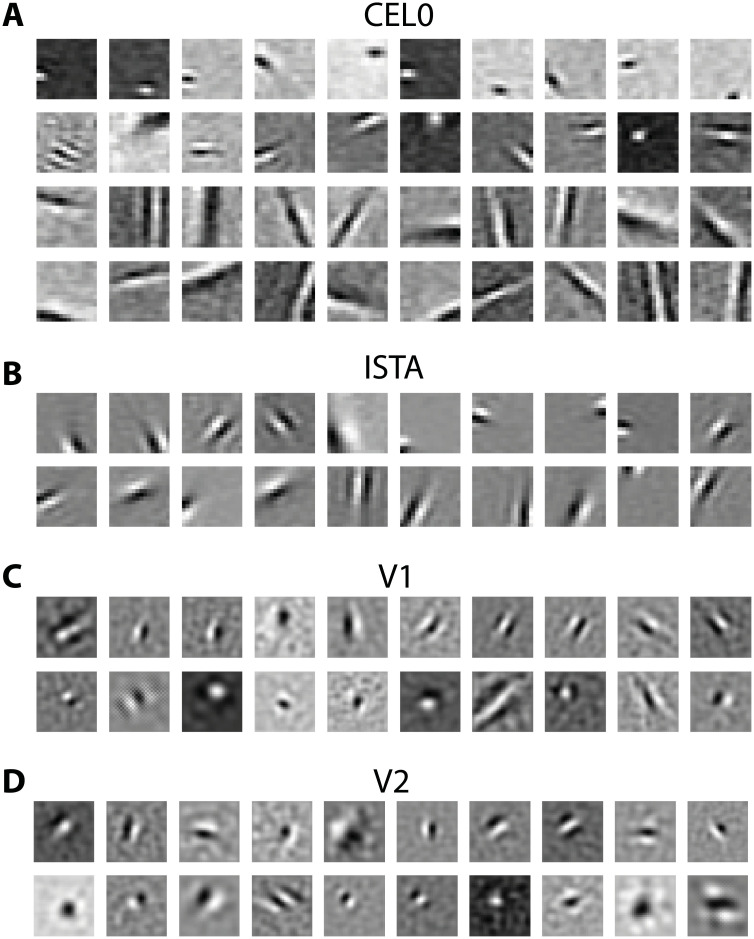
Sampling of RFs of different aspect ratios from our thresholding algorithms and recordings on macaques’ V1 and V2. A: Sampling of RFs generated by CEL0. As we go down the rows their aspect ratio (defined as the width (SD) of the Gaussian envelope parallel to the axis of the Gabor over the width orthogonal to it) increases. The same RF organization applies to the rest of the Figures. B: RFs generated by ISTA. C: RF subunits from electrophysiological recordings in V1. D: Same as (C) for V2 (data courtesy of Liang She).

Visual inspection of the RFs generated by the different thresholding algorithms for 500 units suggests that they are not alike (Fig 6A and 6B show a sampling of the RFs for CEL0 and ISTA, S6, S7, S8 and S9 Figs the full set of 500 units for each algorithm). In particular, we see that CEL0 produces RFs with greater variability of shapes compared to the other methods. Here, we first focus on a comparison between the RFs produced by CEL0 and ISTA, and subsequently with biological neurons.

**Fig 7:**
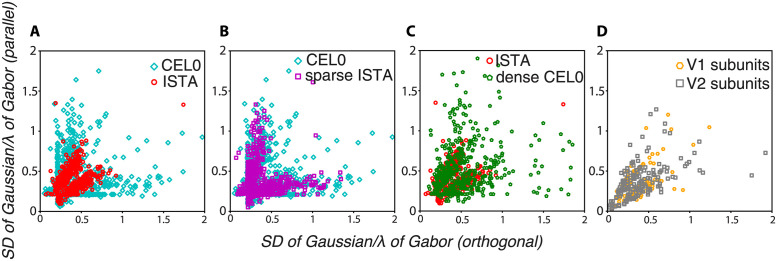
Spatial properties of RFs generated by thresholding algorithms and in macaques’ V1 and V2. A: Width (SD) of the Gaussian envelope along the parallel axis of the Gabor as a function of the width orthogonal to it (both normalized by the Gabor’s period) for the fits of the RFs generated by ISTA and CEL0. B: Same as (A) for CEL0 and an instance of ISTA with the same sparsity level as CEL0 (sparse ISTA). C: Same as (A) for ISTA and an instance of CEL0 with the same sparsity level as ISTA (dense CEL0). D: Same as (A) for the RF subunits from recordings in macaque V1 and V2.

To probe the shapes of the RFs, we fitted the *ϕ* vectors with Gabors using maximum likelihood. We found that the shapes of the RFs as represented by the widths of the Gaussian envelopes along the axes parallel and orthogonal to the gratings showed greater variability for CEL0 compared to ISTA (Fig 7A). CEL0 contained most of its data points near the origin with ISTA having them exclusively there. That region on the graph points to shapes that are either circular or slightly elongated. Visual inspection of the RFs near the origin indicates a distinctive difference between CEL0 and ISTA: CEL0 contains RFs that are reminiscent of the difference of Gaussian filter that typically models retinal and LGN cells (Fig 6A first row), but also found in V1 [33, 53], while ISTA does not (Fig 6B first row). CEL0 also contains RFs with long widths either along the axis parallel or orthogonal to the enveloped grating irrespective of the size of their paired width (Fig 7A). These regions on the graph point to elongated shapes (Fig 6A third and fourth row) mostly absent for ISTA.

**Fig 8:**
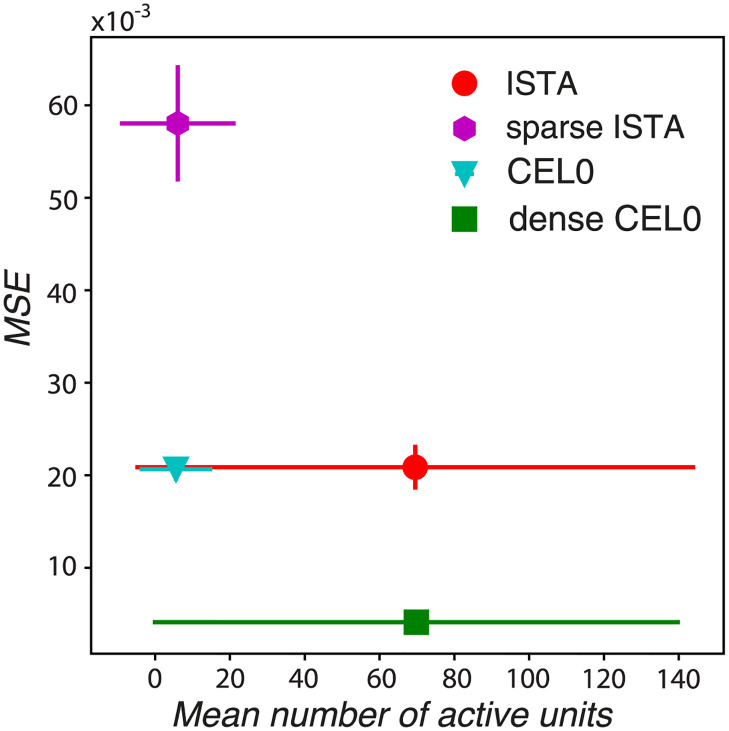
Control for probing the effect of sparsity on the RFs. MSE values computed between the reconstructed and the actual image for the last 500 batches as a function of the mean number of active units for two instances of ISTA and CEL0. Horizontal lines indicate the standard deviation of the mean number of units, vertical the standard deviation of the MSE.

We subsequently ask whether the degree of sparsity is the determining factor in the differentiation of a homogeneous set of RFs into a more variable one with RFs with different functions. To test that, we set the λ parameter of ISTA so that it codes image patches with the same number of active units on average as CEL0, and compare the two sets of RFs. We refer to this choice as sparse ISTA (compare in Fig 8 the abscissas of sparse ISTA and CEL0). We found that, unlike its original instance (Fig 7C), sparse ISTA becomes as variable in its distribution of shapes as CEL0 (Fig 7B). Note that in order to gain this variability, sparse ISTA degraded significantly its reconstruction capacity, having a MSE approximately 3 times worse than the original instance of ISTA and of CEL0 (Fig 8). We also expected that CEL0 would lose the variability in its RFs if it became less sparse. To test that, we set the λ parameter of CEL0 so that on average it codes with the same number of active units as the original instance of ISTA (we call this version dense CEL0; compare in Fig 8 the abscissas of ISTA with dense CEL0). We found that dense CEL0 loses most of its RF variability, but still contains some RF widths away from the origin (Fig 7C). The latter result indicates that the degree of sparsity is a determining factor in the variability of the RFs produced, but there could also be something intrinsic in the algorithms that is a factor as well.

To probe at which sparsity level the generated RFs best fit with the experimental data, we compared them with V1 and V2 RF subunits of macaque monkeys acquired from single-unit recordings while the monkeys performed a simple fixation task and random grayscale natural images were shown [33]. The authors therein used projection pursuit regression to associate each neuron with one or more subunits (filters); the firing rate of a neuron was estimated by a linear-nonlinear model where the image stimulus was passed through each of the subunits associated with the neuron separately. These outputs were then transformed through a nonlinearity and finally summed. We fitted the subunits with Gabors (we excluded subunits that were below a goodness of fit *r*^2^ threshold of 0.6, keeping 119 V1 and 171 V2 subunits; not taking into account V2 subunits showing complex selectivity) and plotted the widths of their Gaussian envelopes to asses their shape. We found that most V1 and V2 subunit widths are located near the origin (Fig 7D) and best fit with the RF shapes of ISTA and dense CEL0 (Fig 7C). For this sparsity level, dense CEL0 has approximately five times better reconstruction performance than ISTA (Fig 8).

We note by visual inspection that a few biological V1 and V2 subunits have a similar shape to retinal ganglion and LGN neurons, best modelled by a difference of Gaussian model (Fig 6C and 6D). Our previous analysis does not capture this type of RF, but we can observe it for both CEL0 (Fig 6A first row) and sparse ISTA. For this sparsity level, CEL0 has approximately three times better reconstruction performance than sparse ISTA (Fig 8). Our analysis shows that even though both *ℓ*_0_ and *ℓ*_1_ based regularization can produce RFs akin to V1 neurons, CEL0 provides a far superior reconstruction performance at any sparsity level and dictionary size. We argue that this robustness is more likely to characterize neural processing in the visual cortex.

## Discussion

We have shown here that continuous, non-convex thresholding-based algorithms produced much sparser activations for the same reconstruction error compared to the classically used soft thresholding algorithm, ISTA, corresponding to the convex *ℓ*_1_ regularizer. When the same level of sparsity for all algorithms is maintained, CEL0 had the best reconstruction performance for all the dictionary sizes tested (from ∼ 2× to ∼ 20× degrees of overcompleteness). Furthermore, to reach the same performance as CEL0, ISTA needed about 10 times more units.

By considering the circular variance measure, we found that all algorithms produce RFs that represent a subset of V1 neurons in terms of orientation tuning sharpness, but as we increased the number of units available, most of them became broader in their orientation selectivity. Thus, in principle, different pools of neurons, each encoding separately the external world, could represent the whole gamut of orientation selectivities found in V1 [53]. This is supported by experimental evidence showing that V1 neurons are divided into several pools with different objectives following diverging visual streams [54–56], with these pools being divided further into different sub-pools, each representing the whole visual space, though there may be some overlap in their populations [57]. Furthermore, all algorithms replicated the cardinal orientation bias found in V1 [26, 27]: they have a disproportionally larger number of units responding to vertical orientations with those units on average being more sharply tuned compared to the rest.

We found that the RF shapes of V1 (and V2 subunits) are contained in both RF sets of ISTA and CEL0 at a specific sparsity level, and are mostly circular or slightly elongated [33]. In a previous computational study [30], Rehn and Sommer found a contrasting result to ours, i.e. a generative model that used soft sparsity (similar to *ℓ*_1_ regularization) with learned RFs whose shapes did not fit well with the ones of V1 neurons recorded from macaques [53]. The authors showed that an improved model enforcing stronger sparsity did. The V1 neurons used in that study [53] had similar width distributions as the V1 (and V2) subunits we used [33], with most of the points being near the origin. We speculate that the discrepancy in the results are either because of differences in the type of optimization considered or because today’s computational resources afforded us to examine a wider range of sparsity levels for each method (by varying the weighting of the regularization controlled by the λ parameter) compared to [30].

Sparsity produces secondary effects in V1, such as orientation selectivity and variability in the RFs of neurons. This appears to be a common strategy in the brain. For example, it is shown computationally that homeostatic processes, which aim to balance the activity in the brain, also generate neural networks that endow context sensitivity to RFs [58], and connectivity patterns with different degrees of specificity, flexibility, and robustness [59].

Efficient coding can be formulated as a generative probabilistic model that aims to describe natural images’ complex probability distributions as linear combinations of the units vectors (RFs) with the weighting of the linear combination given by the vector of coefficients, *r*, representing the underlying causes of an image. This probabilistic formulation yields the same energy function as in (2), with the regularization function on activity (that defines each thresholding algorithm), being interpreted as the prior distributions of activities, *r* [30, 36, 60]. The prior distributions are highly peaked at zero [36] where in non-convex regimes, they change from Laplace-type to Dirac-like [30]. The activations associated to each unit are assumed to be independent of each other in line with Barlow’s proposal that the sensory cortex performs a redundancy reduction operation that results in statistically independent activations of neurons [61]. Another class of models that follows Barlow’s proposal is independent component analysis (ICA) which finds the statistically independent components of natural images yielding localized edge detectors as well [62].

Our iterative thresholding implementations of the coding step are closely related to neurally plausible architectures [63, 64]. More specifically, similarly to these architectures our implementation includes in the gradient step a local competition term, where active units inhibit other units with similar RFs, and an excitatory input current term that is proportional to how well the input image matches with the RF of the unit. The thresholding operation of the internal states in [63, 64] takes the form of the proximal operator in our case. Finally, as in [63, 64], we let the units’ activities charge up from zero. Due to the inhibition term, the iterative coding operation pushes for concurrent activation of units whose features (RFs) yield pairwise inner product values at or close to zero. In accord to our implementation, neural networks with recurrent competition inducing lateral inhibition between units have been shown to be more robust against noisy stimuli and adversarial attacks compared to feedforward topologies [65].

If we assume that units’ activations map monotonically to action potentials and that the number of action potentials is the sole indicator of energy expenditure, then *ℓ*_1_ regularization can be considered optimal in terms of energy efficiency. Thus in principle, if that was the case there would be some kind of load balancing implemented in the brain where many neurons firing at low rates would encode the outside world. In contrast, *ℓ*_0_ regularization corresponds to a regime where encoding of a stimulus happens by few neurons firing vigorously. The load balancing hypothesis implemented by *ℓ*_1_ regularization runs counter to recent results showing that neural communication consumes 35 times more energy than computation in the human cortex [31]. This indicates that neurons must relay sufficient bits per second to other neurons to justify the cost of communication. In this context, *ℓ*_1_ regularization is a very costly communication system since more neurons (compared to *ℓ*_0_ regularization) are employed for encoding and relay of information. Experiments on sensory coding where few neurons firing consistently can represent robustly the stimuli corroborate these results (see [32] for a recent work).

## Supporting information

S1 Fig Examples of raw and whitened images. First row shows examples of raw images from the van Hateren database. Second row shows the corresponding whitened images used in the generative model.
(EPS)

S2 Fig RFs overlaid by their orientation tuning curves. The most frequently used *ϕ* vectors from each thresholding method for the analysis in Fig 2 (500 units) overlaid by their orientation tuning curves.
(EPS)

S1 Table Values of learning rate *r*, *μ*, and relative weight, λ, for the different algorithms for 500 units. The learning rate for Φ, *η*, in all cases is 10^−2^. For all algorithms the learning rates were constant.
(PDF)

S3 Fig All thresholding algorithms show a sharp decrease in MSE after just a few batch iterations. MSE between the reconstructed and the actual image as a function of iterations of batches for the different thresholding algorithms.
(EPS)

S4 FigSparsity and reconstruction error are not affected by the Φ dictionary used for coding.A: (left figure) MSE of the last 500 iterations as a function of the thresholding method when Φ_*ISTA*_ is used as a dictionary. (right figure) Distribution of activity of the units for the image stimuli presented when Φ_*ISTA*_ is used as a dictionary. B: Same as (A) for Φ_*CEL*0_ as fixed dictionary C: Same as (A) for $\Phi_{\ell_{1/2}}$ as fixed dictionary D: Same as (A) for Φ_*Hard*_ as fixed dictionary.(EPS)

S2 Table Values of the λ parameter for the different algorithms and number of units. For ISTA, *ℓ*_1/2_, and hard thresholding, the values for *μ*, are the same for the different number of units and as shown in S1 Table. For these three algorithms, the learning rate is also constant. For CEL0, *μ*, is 0.05 from 500 to 3750 units, and 0.04 afterwards while *η* is 5 × 10^−3^ for all dictionary sizes. For CEL0, the learning rates decay with iterations based on a time-based decay schedule, with decay rates for both learning rates being their initial values divided by 50.
(PDF)

S5 Fig Sparsity was constrained in a narrow window for all the dictionary sizes. Normalized proportion of active units for the different number of units tested. The normalized proportion of active units is defined as the average number of active units for an image patch over the total number of units in the dictionary. The values taken by the parameter was between 0.0105 and 0.0132. Vertical lines indicate standard error from mean.
(EPS)

S6 Fig 500 *ϕ* vectors learned from ISTA. (EPS)

S7 Fig 500 *ϕ* vectors learned from CEL0. (EPS)

S8 Fig 500 *ϕ* vectors learned from *ℓ*_1/2_ thresholding. (EPS)

S9 Fig 500 *ϕ* vectors learned from hard thresholding. (EPS)
