# Supplementary material for: Beyond ℓ1 sparse coding in V1
Source: PLoS Comput Biol. 2023 Sep 12;19(9):e1011459. doi: 10.1371/journal.pcbi.1011459 (PMC10516432; doi:10.1371/journal.pcbi.1011459)
Supplement: S1 Table — The learning rate for Φ, η, in all cases is 10−2. For all algorithms the learning rates were constant. (PDF) [file pcbi.1011459.s003.pdf]

Methods	ISTA	$\lambda_{1/2}$	Hard	CEL0
μ	0.01	0.01	0.01	0.1
λ	0.41	0.13	0.013	0.45
