# Supplementary material for: Beyond ℓ1 sparse coding in V1
Source: PLoS Comput Biol. 2023 Sep 12;19(9):e1011459. doi: 10.1371/journal.pcbi.1011459 (PMC10516432; doi:10.1371/journal.pcbi.1011459)
Supplement: S2 Table — For ISTA, ℓ1/2, and hard thresholding, the values for μ, are the same for the different number of units and as shown in S1 Table. For these three algorithms, the learning rate is also constant. For CEL0, μ, is 0.05 from 500 to 3750 units, and 0.04 afterwards while η is 5 × 10^−3^ for all dictionary sizes. For CEL0, the learning rates decay with iterations based on a time-based decay schedule, with decay rates for both learning rates being their initial values divided by 50. (PDF) [file pcbi.1011459.s006.pdf]

Number of units	λ_{ISTA}	$\lambda_{\ell_{1/2}}$	λ_{Hard}	λ_{CEL0}
500	1.8	0.35	0.046	0.57
750	1.7	0.33	0.043	0.38
1000	1.6	0.31	0.04	0.27
1250	1.5	0.295	0.037	0.21
1500	1.4	0.285	0.034	0.16
1750	1.35	0.275	0.032	0.13
2000	1.3	0.265	0.031	0.11
2250	1.2	0.255	0.029	0.09
2500	1.15	0.245	0.028	0.08
2750	1.1	0.235	0.027	0.07
3000	1.05	0.225	0.026	0.06
3250	1	0.215	0.025	0.05
3500	0.95	0.205	0.024	0.045
3750	0.925	0.2	0.0235	0.045
4000	0.9	0.195	0.023	0.045
4250	0.875	0.19	0.0225	0.04
4500	0.85	0.185	0.022	0.035
4750	0.85	0.185	0.022	0.035
5000	0.85	0.185	0.022	0.03
